# Effects of Selective α7 Nicotinic Acetylcholine Receptor Stimulation in Oligodendrocytes: Putative Implication in Neuroinflammation

**DOI:** 10.3390/cells14130948

**Published:** 2025-06-20

**Authors:** Claudia Guerriero, Giulia Puliatti, Tamara Di Marino, Giulia Scanavino, Carlo Matera, Clelia Dallanoce, Ada Maria Tata

**Affiliations:** 1 Department of Biology and Biotechnologies, Sapienza University of Rome, Piazzale A. Moro 5, 00185 Rome, Italy; claudia.guerriero@uniroma1.it (C.G.); giu.puliatti@gmail.com (G.P.); tamaradimarino@outlook.it (T.D.M.); giulia.scanavino@uniroma1.it (G.S.); 2 Department of Pharmaceutical Sciences, University of Milan, 20133 Milan, Italy; carlo.matera@unimi.it (C.M.); clelia.dallanoce@unimi.it (C.D.); 3 Research Center of Neurobiology “Daniel Bovet”, Sapienza University of Rome, Piazzale A. Moro 5, 00185 Rome, Italy

**Keywords:** α7 nicotinic receptors, oligodendrocytes, cell proliferation, oxidative agents, neuroinflammation

## Abstract

α7 nAChRs are known to modulate several physiological and pathological functions in glial cells, and their selective activation might have anti-inflammatory effects in the central and peripheral nervous system. OL progenitors (OPCs) respond to cholinergic stimuli via muscarinic receptors that are mainly involved in the modulation of their proliferation. Conversely, the role of nicotinic receptors, particularly α7 nAChRs, has been poorly investigated. In this study, we evaluated the expression of α7 nAChRs in a model of OPCs (Oli neu) and the potential effects mediated by their selective activation. Methods: Oli neu cells were used as a murine immortalized OPCs model. The effects of α7 nAChRs stimulation on cell proliferation and survival were assessed by the MTT assay. RT-PCR and Western blot analysis were used to analyze the expression of α7 nAChRs and proliferative and differentiative markers (PCNA, MBP). LPS exposure was used to induce the environment in which the antioxidant and anti-inflammatory properties of α7 nAChRs were analyzed, evaluating NFR2 and TNF-α expression, ROS levels through DCFDA staining while Oil Red O staining was used for the analysis of lipid droplet content as a marker of cellular inflammation response. Results: The α7 nAChR is expressed both in OPCs and OLs, and its stimulation by the selective agonist ICH3 increases cell proliferation without modifying the OLs’ differentiation capability. Moreover, ICH3 showed anti-inflammatory and antioxidant effects against LPS exposure. Conclusions: The results herein obtained confirm the role of α7 nAChR in the modulation of neuroinflammatory processes as well as their protective effects on OLs.

## 1. Introduction

Neuroinflammation is a defensive mechanism activated within the nervous system as a consequence of infections or neurodegeneration, typically associated with neurological disorders [1]. Glial cells such as astrocytes and microglia participate actively in regulating some neuroinflammatory pathways, but the role played by oligodendrocytes (OLs) in this context is still poorly investigated. OLs, the myelinating glial cells of the CNS, are dynamic cells that may contribute to the maintenance of CNS homeostasis [2,3]. In this context, a deeper exploration of the role of OLs in neuroinflammation becomes particularly intriguing.

Several analyses have highlighted that nicotinic acetylcholine receptors (nAChRs) have a new emerging role in regulating neuroinflammation [1]. Evidence of the neuroprotective role of nAChRs was obtained in the rat cerebral cortex, in which the neuroprotective effects were antagonized by mecamylamine, a nAChR antagonist, but not by scopolamine, a muscarinic acetylcholine receptor antagonist [4]. Moreover, some research groups have shown that targeting the α7 nAChR subtype provides neuroprotection in neurons and glial cells. In particular, the stimulation of α7 nAChR in the glial cells triggers the activation of the “cholinergic anti-inflammatory pathway”, primarily associated with the activation of the master regulator of oxidative stress, nuclear factor erythroid 2-related factor (NRF2) and heme oxygenase-1 (HO-1) [5,6,7]. Additionally, α7 nAChR modulates other key regulators of inflammation and oxidative stress, activating different metabotropic pathways such as Jak/STAT3. Janus kinase 2 (Jak) can initiate signal transduction mediated by signal transducer and activator of transcription 3 (STAT3) [8,9,10]. Specifically, STAT3 blocks the translocation of NF-kB into the cell nucleus and the subsequent binding of NF-kB to DNA, negatively regulating the expression of inflammatory genes. Furthermore, phosphorylated STAT3 binds to form a dimer that can translocate into the nucleus and bind to DNA. In the dimer form, STAT3 positively regulates the transcription of the suppressor of cytokine signaling 3 (SOCS3) [1,11]. Moreover, it has been demonstrated that the selective activation of α7 nAChR can reduce TNF-α levels and release cyclooxygenase-2 and prostaglandin E2: these effects are counteracted by α-bungarotoxin, confirming the α7 nAChR implication in the modulation of neuroinflammation [12].

Considerable evidence supports the beneficial properties of α7 nAChRs in several models, but the central question remains in understanding the mechanism by which α7 nAChR mediates neuroprotection. For these reasons, the identification of new ligands able to act as selective agonists of the α7 nAChRs is crucial to investigate their function and to identify new potential diagnostic markers and therapeutic targets.

Based on our previous studies, we investigated the effects of α7 nAChR stimulation with the selective agonist ICH3 [13]. The pharmacological profile of this orthosteric α7 agonist was previously studied in different models, among them in rat oxaliplatin-induced neuropathy, to explore the role of α7 nAChR in pain reduction, nervous tissue protection, and glial activation. The effects of ICH3 were also assessed in obesity models, demonstrating the potential role of this ligand in mediating the α7 nAChR anti-inflammatory pathway [14]. Moreover, the selective activation of α7 nAChRs by ICH3 in peripheral nerve regeneration contributes to improving Schwann cells (SCs) regenerating properties and promoting a supportive microenvironment [15,16].

Based on our previous results, considering the ability of oligodendrocytes (OLs) to respond to neuroinflammatory environments occurring in the nervous system during neurodegenerative and demyelinating diseases and considering the sensitivity of OLs to oxidative agents present as products of neuroinflammation, we tested the protective role of α7 nAChR for OLs. To this aim, in this paper, we planned to evaluate the effects of α7 nAChR stimulation on the modulation of reactive oxygen species (ROS) production and lipid content and its ability to promote OL proliferation and survival.

## 2. Material and Methods

### 2.1. Cell Culture

The Oli neu cells were obtained from immortalized oligodendrocyte precursor cells (OPCs) derived from the brains of 15-day-old mouse embryos. The cell immortalization was obtained by retroviral transfection of the t-neu tyrosine kinase after the removal of neurons by immunocytolysis [17,18]. Oli neu cells were cultured as previously described [19].

### 2.2. Pharmacological Treatments

(*R*)-(-)-3-Methoxy-1-oxa-2,7-diaza-7,10-ethanospiro [4.5]dec-2-ene sesquifumarate (ICH3) was synthesized as previously reported [13]. The agonist was used at a final concentration of 10 μM according to previous results obtained in other cell models [15,16].

Oli neu cells, as OL progenitors (OPCs), display a bipolar morphology. Like OPCs, when cultured in the presence of thyroid hormones, they spontaneously undergo differentiation, assuming a branched morphology, like mature OLs. To assess the effect of ICH3 treatment on both levels of differentiation, experiments were performed at two time points, 24 h after treatment and 120 h after treatment, in order to evaluate the ICH3 both in OPCs and in more differentiated OLs. Oli neu cells were exposed to lipopolysaccharide (LPS, Sigma-Aldrich, St. Louis, MO, USA) at a final concentration of 1 μg/mL in order to induce an inflammatory environment in vitro. The choice to use LPS is dependent on its potent ability to stimulate inflammation. In particular, it is involved in several pathologies of the central and peripheral nervous system. For example, in sepsis conditions, LPS can cross the blood–brain barrier and cause acute brain inflammation, leading to encephalopathy and cognitive alterations. Moreover, chronic inflammation induced by LPS may contribute to the degeneration of dopaminergic neurons in the substantia nigra, favoring the development of Parkinson’s disease, or worsen myelin damage in multiple sclerosis. Moreover, systemic inflammation, stimulated by LPS, has been associated with neurochemical and behavioral alterations that may contribute to depression and anxiety [20].

α-bungarotoxin (α-BTX) at 100 nM concentration was used as a selective antagonist of α7 nAChRs [16,21].

### 2.3. Cell Viability Assay

The colorimetric assay based on 3-(4.5-dimethyl thia-zol 2-y1)-2.5-diphenyl tetrazolium bromide (MTT, Sigma-Aldrich, St. Louis, MO, USA) metabolization was used to assess cell proliferation, according to the protocol optimized by Mosmann [22]. This assay was performed on Oli neu cells cultured on poly-l-lysine-coated 96-well plates at a density of 1.2 × 10^4^ cells/well. After 24 h from seeding, cells were treated for different experimental times (ranging from 1 to 4 days in vitro; DIV) with 10 μM ICH3, 100 μM muscarine, 100 nM α-BTX, α-BTX + ICH3; the control is represented by the untreated cells, kept in culture in complete medium without addition of other factors.

Muscarine was used as a control since this molecule is known, from previous studies, to stimulate cell proliferation of oligodendrocyte precursors (OPCs) [23].

The MTT assay was performed as previously described [19]. Briefly, MTT at proper concentration use was added to each well and, after 3 h incubation, the dark blue crystals were mechanically dissolved with the addition of a solution made up of isopropanol, HCl 0.04 M, and 1% Triton X-100. Multiskan FC (Thermofisher Scientific, Waltham, MA, USA). The optical density (OD) at 570 nm of each well was measured by Multiskan FC (Thermofisher Scientific, Waltham, MA, USA).

The trypan blue staining was performed as a viability assay in order to assess any potential toxicity of ICH3 and LPS on Oli neu cells. After the treatment with 10 μM ICH3, 1 μg/μL LPS + DMSO, ICH3 + LPS for 24, 72, and 120 h, Oli neu cells were collected and incubated with trypan blue, which can penetrate and thus stain only dead cells. LUNA-FX7TM Automated Cell Counter (Logos Biosystems, Anyang, Republic of Korea) was used to measure the percentage of dead cells.

### 2.4. Morphological Analysis

Oli neu cells were plated onto 60 mm dishes coated with poly-l-lysine at a density of 3.5 × 10^5^ cells/well. After one day, cells were treated with 10 μM ICH3. After 72 h and 120 h post-treatment, 3 bright-field images/dish/each experimental condition were acquired. The number of processes emitted by each cell was measured using ImageJ 1.53 imaging software (NIH, Bethesda, MD, USA). The data collected were obtained from three independent experiments.

### 2.5. Total RNA Extraction and RT-PCR Analysis

RNA extraction from Oli neu cells was performed using TRI Reagent^®^ (Sigma-Aldrich, St. Louis, MO, USA), according to the manufacturer’s instructions. After quantification, 1 μg of total RNA for each sample was reverse transcribed, and 100 ng of the resulting cDNA was amplified through polymerase chain reaction as previously described [19]. The sequences of the primers used are *a7 nAChR:* forward 5′-AACCATGCGCCGTAGGACA-3′; reverse 5′-CTCAGCCACAAGCAGCAGCATGAA-3′.

*GAPDH*: forward 5′-TGGCATTGTGGAAGGGCTCATGA-3′; reverse 5′-ATGCCAGTGAGCTTCCCGTTCAG-3′.

### 2.6. Protein Extraction and Western Blot Analysis

Protein extraction was performed by cell homogenization in lysis buffer (Tris-EDTA 10 mM, 0.5% NP40, and NaCl 150 mM) supplemented with a protease inhibitor cocktail (Sigma-Aldrich, St. Louis, MO, USA). The concentration of protein lysates was quantified with a Pierce BCA Protein Assay Kit (Thermo Fisher Scientific, Waltham, MA, USA) according to the manufacturer’s protocol. Western blot analysis was carried out as described in ref. [19]. Briefly, protein samples were separated on SDS-polyacrilamide gel (SDS-PAGE) and transferred to polyvinylidene difluoride (PVDF) membrane (Merck Millipore, Darmstadt, Germany). After blocking, membranes were incubated with one of the following primary antibodies: anti-PCNA (dilution 1:700, Sigma-Aldrich, St. Louis, MO, USA), anti-α7 nAChR antibody (dilution 1:800, Bioss, Woburn, MA, USA), anti-NRF2 (1:1000, Immunological Science, Milan, Italy); anti-α tubulin (dilution 1:500, Immunological Science, Milan, Italy) and anti-Histone H3 (1:5000, Abcam, Cambridge, UK) were used as loading controls. Anti-rabbit horseradish peroxidase (1:10,000, Promega, Madison WI, USA) or anti-mouse horseradish peroxidase (1:10,000, Immunological Science, Milan, Italy) were appropriately used as secondary antibodies. ECL chemiluminescence reagents (Immunological Science, Milan, Italy) revealed the immunoreaction, and the signal detection was obtained with Chemidoc (Molecular Imager ChemiDoc XRS + System with Image Lab Software; Bio-Rad, CA, USA).

### 2.7. ROS Detection Assay

The ROS detection assay was performed on Oli neu cells cultured in 96-well plates with poly-l-lysine coating at a density of 1 × 10^4^ cells/well and treated with 10 μM ICH3, 1 μg/μL LPS, ICH3 + LPS, for 2 h, 20 μM. H_2_O_2_ was used as positive control. The ROS production was measured exploiting the *N*-acetyl-l-cysteine (NAC; Enzo Life Sciences, New York, NY, USA) as ROS scavenger as well as the dichloro-dihydro-fluorescein diacetate (DCFDA; Enzo Life Sciences, New York, NY, USA) as an oxidation substrate [19]. The fluorescence was read at λ = 485 nm of excitation and λ = 530 nm of emission by the Glomax Multi Detection System (Promega Italia, Milan, Italy).

### 2.8. Lipid Droplets Detection Assay

The Oil Red O was the fat-soluble dye used to detect lipid droplets (LDs), being able to turn them orange-red color. For LDs detection assay, Oli neu cells were seeded on poly-l-lysine-coated 24-well plates at a density of 3 × 10^4^ cells/well and treated with 10 μM ICH3, 1 μg/μL LPS, ICH3 + LPS. After 24 h or 120 h from the treatment, the cells were fixed with 4% paraformaldehyde for 20 min at RT and carefully washed in PBS. The staining with the Oil Red O:dH2O solution (*v*/*v*, 2:3) and Hoechst 33342 was performed as described in [19]. A Zeiss microscope with the Zen lite 3.7 software (Zeiss, Oberkochen, Germany) was used for the image acquisitions.

The absorbance measurement of Oil Red O staining also provided a quantitative analysis according to a previously described procedure [19]. In this case, Oli neu cells were seeded on poly-l-lysine-coated 96-well plates at a density of 1 × 10^4^ cells/well, treated as above for 24 h or 120 h, and then incubated with the same working solution (Oil Red O:dH2O *v*/*v*, 2:3) for 30 min in the dark. After washing and adding isopropanol, the OD at 570 nm was measured by Clariostar Plus Microplate Reader (BMG Labtech, Ortenberg, Germany) in order to quantify LD production.

### 2.9. Statistical Analysis

Data were presented as the mean ± standard error of the mean (SEM). Student’s *t*-test or one-way ANOVA tests were used to evaluate statistical significance within the different samples. Results were considered statistically significant at *p* < 0.05 (*), *p* < 0.01 (**), *p* < 0.001 (***), and *p* < 0.0001 (****). Data analyses were performed with GraphPad Prism 9 (GraphPad Software, La Jolla, CA, USA).

## 3. Results

### 3.1. Analysis of α7 nAChR Expression

**Figure 1:**
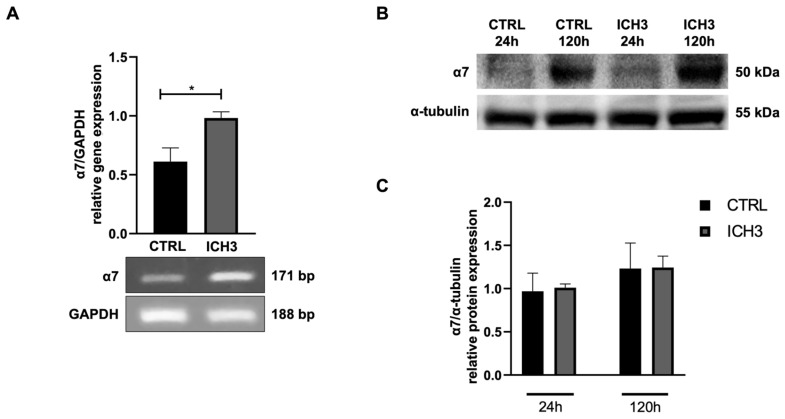
(**A**) RT-PCR analysis of α7 nAChR transcript levels in Oli neu cells in CTRL or upon 24 h of 10 µM ICH3 treatment. GAPDH was used as the housekeeping gene. The gel reported is representative of three independent experiments. Densitometric analysis was obtained from the mean ± SEM of three independent experiments. Student’s *t*-test was used to statistically compare the different experimental conditions (* *p* < 0.05). (**B**,**C**) Western blot analysis of α7 expression in Oli neu cells in CTRL or upon 24 h or 120 h of 10 µM ICH3 treatment. α-Tubulin was used as an internal reference protein. The figures are representative of three independent experiments. Densitometric analysis was obtained from the mean ± SEM of three experiments. Student’s *t*-test was used to statistically compare the different experimental conditions (n.s, *p* > 0.05 ctrl vs. ICH3 at 24 h and 120 h).

First, we have analyzed the expression of α7 nAChR after ICH3 agonist treatment in Oli neu cells. ICH3 treatment increases the expression of α7 nAChR transcript (Figure 1A) but not the protein (Figure 1B,C). Interestingly, α7 nAChR expression progressively increases during the maintenance of OPCs in vitro.

### 3.2. Analysis of Cell Proliferation and Morphology

**Figure 2:**
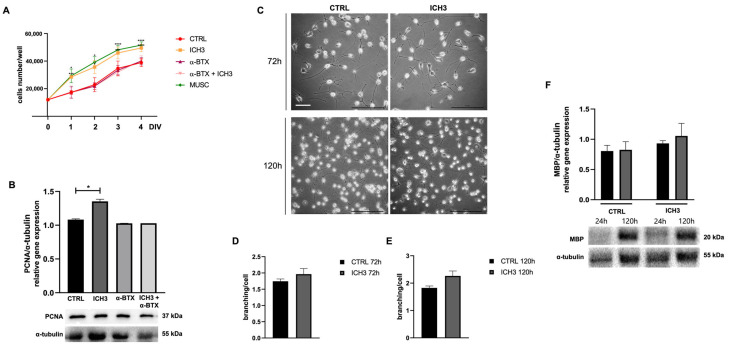
(**A**) Analysis of Oli neu cells growth by MTT assay in the control condition (CTRL, culture medium) and after treatment with 10 µM ICH3, 100 nM α-BTX, 100 nM α-BTX + 10 µM ICH3, and 100 µM MUSC (1, 2, 3, 4 DIV; days in vitro). Data are presented as mean ± SEM of three independent experiments performed in quadruplicate. Student’s *t*-test was used to statistically compare the vehicle and treatment conditions at each different experimental time (* *p* < 0.05, *** *p* < 0.001, **** *p* < 0.0001). (**B**) Representative Western blot for PCNA protein expression in Oli neu cells treated for 72 h as follows: CTRL (culture medium), 10 µM ICH3, 100 nM α-bungarotoxin, and 100 nM α-bungarotoxin + 10 µM ICH3. α-Tubulin was used as an internal reference protein. The densitometric analysis shown in the graph was obtained from three independent experiments. Student’s *t*-test was used to statistically compare the different experimental conditions (* *p* < 0.05). (**C**) Representative images of Oli neu cells after 72 h or 120 h in the control condition or after 10 µM ICH3 treatment. Scale bar: 40 µm. (**D**,**E**) The graphs report the number of branching/cells in the different experimental conditions at 72 h and 120 h of treatment, respectively. Number of cells analyzed at 72 h = 201 (in CTRL condition) and 138 (with ICH3 treatment); number of cells analyzed at 120 h = 722 (in Ctrl condition) and 643 (in the presence of ICH3). Student’s *t*-test was used to statistically compare CTRL and ICH3-treated cells at 72 h of treatment. (**F**) Representative Western blot for MBP expression in Oli neu cells maintained for 24 h or 120 h in culture in the absence or presence of 10 µM ICH3. α-Tubulin was used as reference protein. The graph reported the densitometric analysis obtained from three independent experiments. No significant difference was observed in CTRL and ICH3 conditions at both time points analyzed.

The MTT assay is commonly used to evaluate cell growth [23,24]; for this purpose, it was used to examine the possible effects of ICH3 on Oli neu cell growth. We evaluated the effect of ICH3 on Oli neu cells after 1-2-3-4 DIV (day in vitro) of treatment, comparing the following experimental conditions: 10 µM ICH3, 100 nM α-BTX, 100 nM α-BTX + 10 µM ICH3, and 100 µM muscarine (MUSC); the control condition consisted of Oli neu cells grown in standard culture medium in the absence of cholinergic agonists or antagonists. The α-BTX is a known antagonist of α7 nAChR [25] and, therefore, was used to evaluate its ability to prevent the ICH3 effects. Muscarine was used as a positive control, given that previous studies [23] demonstrated that muscarinic stimulation induced the proliferation of oligodendrocyte precursors. We found that ICH3 treatment, as well as muscarine, significantly increased cell growth compared to the control condition (Figure 2A). As expected, the pro-proliferative effects of ICH3 were counteracted by α-bungarotoxin, which did not show significant changes when used alone (Figure 2A).

This result was further supported by the increase in Proliferating Cell Nuclear Antigen (PCNA) protein expression in Oli neu cells treated with 10 µM ICH3 for 72 h, as indicated by Western blot analysis (Figure 2B). Indeed, PCNA is specifically expressed in actively replicating cells; it is a cofactor of DNA polymerase δ and increases the synthesis of the guide strand during DNA replication [26]. Even in this case, α-bungarotoxin alone had no significant effect on PCNA protein expression level compared to the control condition and fully reverted the ICH3 pro-proliferative action when used in combination with the α7 agonist (Figure 2B).

To evaluate the effect of ICH3 treatment on Oli neu cell morphology, the reorganization of the cytoskeleton leading to the extension of cellular processes was investigated, as previously described [19]. After 72 h and 120 h of treatment with 10 µM ICH3 or vehicle (culture medium, ctrl), the morphology of Oli neu cells was analyzed by counting the number of processes emitted by each cell in both experimental conditions (Figure 2C). Oli neu cells, as well as the OPCs, underwent a progressive and spontaneous differentiation if cultured in the presence of thyroid hormones [27]. In line with this outcome, we observed a progressive increase in the number of processes in the control condition, comparing Oli neu cells at 72 h and 120 h of culture. Instead, no significant difference in Oli cell processes branching after ICH3 treatment was observed neither at 72 h nor at 120 h compared to the respective control conditions (Figure 2C–E). In order to verify the putative effects of ICH3 on OL differentiation, we evaluated the expression of Myelin Basic Protein (MBP), a typical myelin protein expressed by Ols, by Western blot analysis. As reported in Figure 2F, the MBP is expressed at very low levels in OPCs at 24 h of culture. However, as expected, its expression increases after 120 h of culture, confirming a spontaneous differentiation of Oli neu cells when maintained in culture in a complete medium. Interestingly, the agonist ICH3 did not modify the MBP expression, either at 24 or 120 h of culture.

### 3.3. Analysis of the Antioxidant Properties Mediated by α7 nAChR Activation

**Figure 3:**
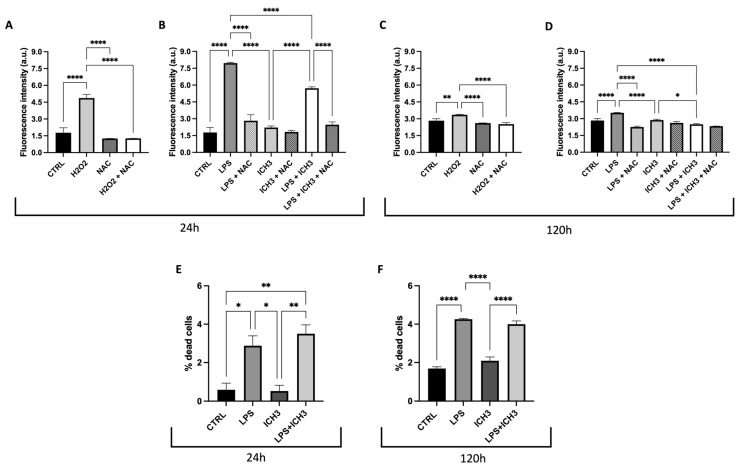
Antioxidant properties mediated by α7 nicotinic agonist in Oli neu cells. Measurement of ROS levels by DCFDA staining in Oli neu cells under the following experimental conditions: 1 μg/mL LPS, 10 μM ICH3, and 1 μg/mL LPS + 10 μM ICH3, in the presence or absence of NAC (10 μM). Cells were analyzed after 24 h (**B**) or 120 h (**D**) from seeding to evaluate the responses of OPCs or pro-OL cells in different experimental conditions. H_2_O_2_ (20 μM) treatment was a positive control both in cells maintained in culture for 24 h (**A**) and 120 h (**C**). Data are presented as mean ± SEM of five independent experiments performed in sextuplicate. One-way ANOVA test, followed by the Turkey multiple comparison post-test, statistically compared the different experimental conditions (* *p* < 0.05; ** *p* < 0.01, **** *p* < 0.0001). Cell viability in Oli neu cells after LPS and ICH3 treatment. Oli neu cells were plated in sextuplicate and treated with complete medium (CTRL), ICH3 10 μM, LPS 1 μg/mL, and LPS + ICH3. After 24 h (**E**) and 120 h (**F**) of treatment, the cells were counted using trypan blue dye. The graph, showing the percentage of dead cells, was obtained from the average ± SEM of three independent experiments.

The antioxidant properties of ICH3 have been previously demonstrated [14,15], as well as the specific increase in reactive oxygen species (ROS) intracellular levels induced by LPS [28,29]. Figure 3 shows the effect of ICH3 treatment analyzed on LPS-stimulated Oli neu cells after 24 h and 120 h in culture. The intracellular ROS production was evaluated by dichloro-dihydro-fluorescein diacetate (DCFDA) staining, and the ROS scavenger *N*-acetyl-l-cysteine (NAC) was used for counteracting the ROS increase. As expected, LPS treatment strongly enhanced ROS levels also in Oli neu cells thanks to the Toll-like receptor 4 expression [3,30]. The ROS increase was observed at both the experimental time points considered (24 h and 120 h) as representative of two different stages of OL differentiation (Figure 3B,D). Instead, in the presence of both NAC and ICH3, the LPS-induced ROS levels were significantly reduced. On the other hand, as a positive control of the experiment, the Oli neu cells were also treated with H_2_O_2_ (20 µM), which is known to trigger oxidative stress [31]. As shown in Figure 3A,C, after 24 h and 120 h, respectively, H_2_O_2_ caused a significant increase in ROS levels, albeit to a lower extent than in LPS-treated cells. NAC was able to counteract the H_2_O_2_-induced ROS increase.

To establish if nAChR stimulation has effects on cell viability after LPS treatment, we evaluated the percentage of cell death after 1 μg/mL LPS in the presence or absence of 10 μM ICH3 after 24 h and 120 h of culture. As shown in Figure 3, the treatment with LPS induces an increase in the percentage of dead cells both at 24 h and 120 h (Figure 3E,F). When added alone, ICH3 does not induce significant variation in cell death and, in combination with LPS, does not modify the percentage of LPS-induced dead cells, both in OPCs (at 24 h of culture) and in more differentiated OLs (120 h).

**Figure 4:**
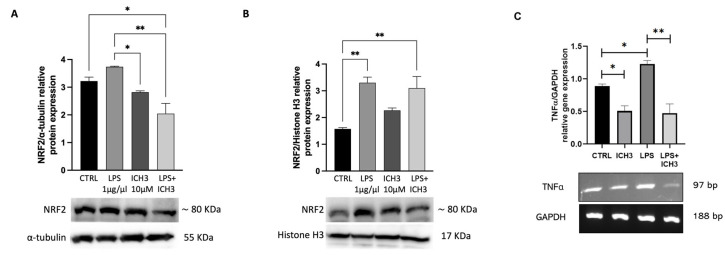
Representative Western blot for protein expression of NRF2 in (**A**) cytoplasmic and (**B**) nuclear extracts of Oli neu cells after 4 h of treatment under the following experimental conditions: CTRL (untreated cells), 1 μg/mL LPS, 10 μM ICH3, and 1 μg/mL LPS + 10 μM ICH3. α-Tubulin and Histone H3 were used as cytoplasmic and nuclear reference proteins, respectively. (**C**) RT-PCR analysis of TNFα transcript levels in Oli neu cells in CTRL or upon 24 h with 1 μg/mL LPS, 10 μM ICH3, and 1 μg/mL LPS + 10 μM ICH3 treatment. Gapdh was used as the housekeeping gene. The gel reported is representative of three independent experiments. Densitometric analysis shown in the graphs was obtained from three independent experiments ± SEM. One-way ANOVA test, followed by the Tukey multiple comparison post hoc test, was used to statistically compare the different experimental conditions (* *p* < 0.05; ** *p* < 0.01).

Considering that the antioxidant and anti-inflammatory system is regulated by Nrf2/Keap1 [32], protein expression levels of Nrf2 were analyzed by immunoblotting at 4 h after treatment with LPS and ICH3. As reported in Figure 4A, a decrease in Nrf2 protein was observed in cytoplasmic extracts of cells treated with ICH3 in the presence of LPS. Under oxidative stress, Nrf2 is phosphorylated and moves from the cytoplasm to the nucleus by disrupting the complex with Keap1 [33,34]. Thereby, Nrf2 translocation was analyzed by Western blot in nuclear extracts obtained by the same samples used to evaluate the Nrf2 cytoplasmic expression. The results showed that, in the CTRL condition, Nrf2 is largely expressed in the cytoplasm, and lower levels are present in the nuclei. In the presence of the LPS, the levels of NRF2 in the cytoplasm and nuclei are comparable, while when ICH3 is present with the LPS, the amount of Nrf2 in the cytoplasm decreases with simultaneous increases in the nuclei (for the control of the cytoplasmic and nuclear protein extraction, see Supplementary Figure S1).

To confirm the anti-inflammatory role of the α7 receptor, we have also evaluated the expression levels of the pro-inflammatory cytokine TNF-α in the same experimental conditions. The results indicate the ability of LPS to increase the TNF-α transcript levels in Oli neu cells. Interestingly when the α7 receptor was stimulated by the ICH3 agonist, we observed a significant decrease in TNF-α mRNA, confirming the anti-inflammatory effects mediated by the α7 receptor (Figure 4C).

### 3.4. Analysis of ICH3 Effects on Lipid Content in Oli Neu Cells

The role of lipid droplets (LDs) in lipid metabolism is well known, but in recent years, these organelles have also become relevant in the cellular stress response, including inflammation. In fact, the stress condition derived from inflammation may contribute to inducing lipid droplet accumulation as metabolic storage for the cells. However when the lipid droplets remain for a long time, they can cause lipotoxicity and contribute to the exacerbation of the damage induced by neuroinflammation [35].

**Figure 5:**
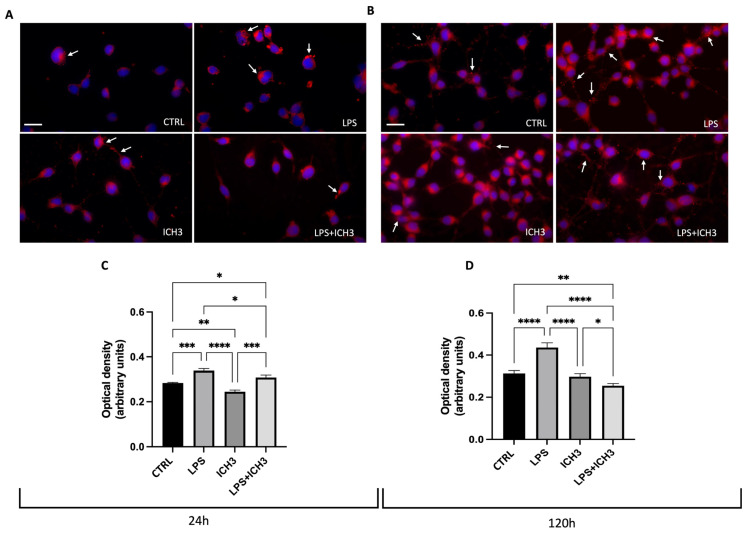
Oil red O staining (in red) in Oli neu cells after (**A**,**C**) 24 h and (**B**,**D**) 120 h from seeding with 1 μg/mL LPS, 10 μM ICH3, and 1 μg/mL LPS + 10 μM ICH3. Images were acquired using a Zeiss fluorescence microscope. For nuclei staining, Hoechst 33342 was used (in blue). Scale bars = 10 μm. The graphs show the optical density relative to the LDs intracellular quantity measured in Oli neu cells in the following experimental conditions: CTRL (untreated cells), LPS, ICH3, and LPS + ICH3, after 48 h (**C**) and 120 h (**D**). Quantification was obtained using a multimodal microplate reader (CLARIOstar^®^ Plus, BMG Labtech). The data are the average ± SEM of three independent experiments performed in sextuplicate. One-way ANOVA test followed by the Turkey multiple comparison post-test was used to statistically compare the different experimental conditions (* *p* < 0.05; ** *p* < 0.01; *** *p* < 0.001; **** *p* < 0.0001).

Considering the oxidative stress observed after LPS stimulation and the ability of α7 stimulation to counteract the inflammation, we have evaluated if, in these experimental conditions, the OPCs and OLs could show a lipid accumulation in lipid droplets. Therefore, we evaluated the content of LDs in Oli neu cells after inflammatory stimulation (LPS treatment) in the presence or absence of 10µM ICH3 at 48 h (Figure 5A,C) and 120 h (Figure 5B,D). Cells were treated with LPS 1 μg/mL, ICH3 10 μM or LPS + ICH3 10 μM for 24 h and 120 h to compare the amount of LDs in Oli neu cells at different stages of OL differentiation by staining with Oil Red O. Through qualitative and quantitative analysis, we observed that, as expected, the amount of LDs was increased after LPS treatment. ICH3 alone did not lead to large changes compared to the untreated cells. After 24 h, ICH3 was able to reduce LD levels in the presence of LPS (Figure 5C), albeit the effects were more evident in pro-OLs (120 h in culture) (Figure 5D).

## 4. Discussion

Whereas the ability of OL progenitors (OPCs) to respond to cholinergic stimuli via muscarinic acetylcholine receptors is well known [36], the role of nAChRs is still poorly understood. In the present study, we assessed the expression of α7 nAChRs in the Oli neu cell line, a representative murine OPCs in vitro model, and investigated the potential effects of their selective activation to potentially understand the role that these receptors might play in OLs in normal and pathological conditions. To this end, we took advantage of ICH3, a selective α7 nAChR agonist prepared and formerly tested by our research group, as an example of its anti-inflammatory properties in various cell models, among which rat Schwann cells [15,16]. Indeed, it has been demonstrated that α7 nAChR is involved in the cholinergic anti-inflammatory pathway, enhancing the production of anti-inflammatory cytokines and suppressing the activity of mononuclear phagocytes, such as dendritic cells and macrophages. As previously demonstrated, Oli neu cells express O4 and Claudin-11, two OLs’ markers [19], and progressively differentiate if cultured in thyroid hormones-supplemented medium [27]. Therefore, all the experiments performed in this study were carried out over time points ranging from 24 h to 120 h to evidence any potential difference related to the differentiation state of Oli neu cells.

The analysis of α7 nAChR expression following ICH3 agonist treatment in Oli-neu cells revealed intriguing results regarding the regulation of this receptor at the transcript and protein levels. Our findings indicate that ICH3 treatment leads to a significant upregulation of α7 nAChR mRNA expression, suggesting a selective enhancement of the transcriptional activity. Notably, despite the increased mRNA levels, this upregulation does not translate into a corresponding increase in protein expression (Figure 1), albeit the increased mRNA levels at 24 h may promote the increased protein expression observed at 120 h. However, a progressive increase in α7 nAChR expression was also observed during the maintenance of OPCs in vitro, suggesting a time-dependent regulation of this receptor during OLs maturation. The absence of differences between protein levels in untreated and ICH3-treated cells at 120 h suggests that potential post-transcriptional or translational regulatory mechanisms may limit the production or stability of the α7 nAChR protein. The lack of a parallel increase in protein expression also points to the possibility that feedback mechanisms within the cells may modulate receptor protein levels, preventing an excessive accumulation that could alter cellular homeostasis.

We also demonstrate that ICH3 induced a significant increase in Oli neu cell growth, strengthening the pro-proliferative effect mediated by muscarine, as also known from a previous study [25]. The proliferation rate was studied by the MTT assay at different experimental times ranging from 1 to 4 DIV. According to the MTT results, we found an increased expression of PCNA in ICH3-treated cells. Indeed, PCNA is predominantly expressed in proliferating cells and plays a crucial role in DNA replication and repair. By contrast, the treatment of Oli neu cells with the α7 nAChR antagonist α-BTX completely counteracted the ICH3 pro-proliferative effect as well as the PCNA increase, confirming that these effects are largely dependent on the selective activation of α7 nAChRs (Figure 1) [4].

However, this boost in cell growth does not correlate with an acceleration of Oli neu differentiation, as assessed by the mean number of cellular processes and the level of MBP expression, a typical myelin protein localized in mature OLs. In fact, we did not find any differences in cell branching between ICH3-treated and untreated cells, neither at 72 h nor at 120 h of culture. On the contrary, we detected a significant overall trend in cell processing increase comparing the two different time points of culture, thus confirming the capability of Oli neu to progressively differentiate in the presence of thyroid hormones. Accordingly, also the MBP expression in Oli neu cells (both in ICH3-treated cells and in control samples) significantly increased from 24 h to 120 h in culture, albeit it was not influenced by ICH3 (Figure 2). Considering the strategic role played by α7 nAChRs in modulating the “cholinergic anti-inflammatory pathway”, we have also evaluated its potential protective effects on OPCs and OLs in inflammatory conditions, a state typically present in the CNS in the presence of neurodegenerative or demyelinating diseases.

OLs are highly susceptible to oxidative stress, and the antioxidant properties of ICH3 have already been demonstrated [14,15]. Our previous studies indicated that OPCs, as well as Oli neu cells, can respond to LPS treatment by engaging TLR4, which activates the transcription of pro-inflammatory cytokines, including IL-6 and TNF-α [3,30]. As indicated above, the choice of using LPS is dependent on its potent ability to stimulate inflammation and relevantly contribute to the promotion or exacerbation of CNS pathologies [20].

Given the connection between inflammatory response and ROS production, we tested the effects of ICH3 treatment after exposure of Oli neu cells to LPS, resulting in an in vitro inflammatory microenvironment. We measured the intracellular ROS levels in the ICH3-treated and untreated cells. As expected, we found that LPS (as well as H_2_O_2_) significantly increased ROS levels in Oli neu cells and that this inflammatory action was counteracted by the ROS scavenger NAC. We also found that ICH3 was able to reduce the LPS-induced ROS levels, substantiating its known antioxidant properties (Figure 3). Moreover, we observed that α7 nAChR agonist was also able to regulate the activation of Nrf2, as proved by the Nrf2 translocation from the cytoplasm to the nuclei of the Oli neu cells. In fact, the early stimulation of Oli neu cells with LPS caused a significant increase in Nrf2 both in the nuclei and in the cytoplasm, but interestingly, the presence of ICH3, both alone and in combination with LPS, caused a significant increase in Nrf2 in the nuclei, suggesting an activation of the antioxidant agent following LPS stimulation (Figure 4). Interestingly we also demonstrated that the expression of pro-inflammatory cytokine TNF-α was significantly increased by LPS, and this increase was countered when ICH3 was added to the culture medium. This result supports the evidence that the ICH-3 counteracts neuroinflammation via NRF2 activation and pro-inflammatory cytokine reduction.

Since we had previously demonstrated that LPS has an effect on cell death in Oli neu cells [19], we examined whether ICH3 was able to counteract the LPS effects. We analyzed the cell death after LPS stimulation with or without ICH3. Interestingly, we confirmed that LPS significantly increased cell death both in OPCs (1 DIV) and in more differentiated OLs (4 DIV); however, α7 nAChR stimulation by ICH3 did not induce cell death and the combined administration of ICH-3 with LPS did not counteract the LPS-mediated effects.

Lipid homeostasis plays a strategic role during neuroinflammation. Lipids are accumulated in LDs, dynamic intracellular organelles that contribute to cell metabolism and have a protective role from many cellular stressors, including oxidative stress. During neurodegenerative disease, LDs are produced and used as lipid reserves useful to support cell metabolism in stressed cells. When the inflammation increases as a consequence of the pathology, the accumulation of lipids becomes, itself, the cause of additional inflammation, contributing to the exacerbation of the cell damage rather than ameliorating it [37]. Considering their involvement in oxidative stress conditions, we evaluated LD accumulation using Oil Red O staining in Oli neu cells in inflammatory conditions in the presence of LPS and in the presence of ICH3 treatment. As expected, quantitative and qualitative analyses revealed increased LD accumulation following LPS exposure, an effect that was significantly reduced by ICH3 treatment at 48 h and, even more evidently, at 120 h of culture. Lipid accumulation after LPS is probably dependent on cellular response to the induced stress, aiming to provide an energy reserve for the OLs in response to the stress. This accumulation is more evident at 120 h when the Oli-neu results in phase of differentiation with greater production of plasma membranes in preparation to possible myelination events. Treatment with ICH3 significantly reduces lipid accumulation after LPS treatment but not respect to control condition. This suggest that this decrease was a consequence of the overall reduction of the inflammatory state, in agreement with previous results demostrating a decrease of oxidative stress, reduced levels of TNFα, and NFR2 translocation into the nuclei. Therefore, in general, the reduction of lipids induced by ICH3 should not have an impact on myelination, as also suggested by the morphological analysis of the cells and the MBP expression reported in Figure 2, which demonstrated that ICH3 stimulation does not alter the spontaneous differentiation process of OLI-neu cells in vitro.

Collectively, our data show that the selective activation of α7 nAChR is able to decrease both the ROS levels and the amount of LDs induced by the inflammatory stimulus of LPS, probably through the NRF2 regulator factor activation and TNFα level reduction. In general, the antioxidant and anti-inflammatory effects of ICH3 were more pronounced after 120 h of culture as a possible consequence of the increased levels of α7 nAChR expression in more differentiated OLs.

## 5. Conclusions

The results discussed in this paper confirm that OLs respond to cholinergic stimuli through both muscarinic and nicotinic receptors. Considering the ability of both receptors to improve OPC proliferation, it is possible to hypothesize that the cholinergic stimuli are required for the recruitment of OPC in physiological as well as pathological conditions. However, OLs are also capable of responding to the inflammatory microenvironment, contributing to modulating the neuro-inflammatory process. The presence of α7 nAChRs in OPCs and OLs might exert a protective role for these cells, particularly during inflammatory events. In fact, we show that the selective activation of α7 nAChRs counteracts the ROS-LPS induced and LDs production; moreover, it promotes Nrf2 translocation in the nuclei and a consequent decrease in the TNF-α expression. Thus, our data suggest a potential role of α7 nAChRs in the reestablishment of CNS homeostasis and in reducing neuroinflammation. Moreover, considering the ability of α7 nAChRs to promote OPC proliferation without modifying the spontaneous ability of OPCs to differentiate into pro-OLs, these effects may have a strategic role to stimulate the production of new oligodendrocyte precursors able to rescue the potentially lost OLs during inflammatory processes. These and other previous results confirm the protective role of α7 nAChRs for OLs and further highlight their ability to modulate the “cholinergic anti-inflammatory pathway” also involving OLs.

## Data Availability

The original contributions presented in this study are included in the article/Supplementary Material. Further inquiries can be directed to the corresponding author.

## Supplementary Materials

The following supporting information can be downloaded at: https://www.mdpi.com/article/10.3390/cells14130948/s1, Figure S1: (A) Representative Western blot showing the faint expression of Histone H3 protein in the cytoplasm extracts of Oli neu cells, to compare with the nuclear extracts in Figure 4B. The relative molecular weight is on the right. (B) Representative Western blot shows the absence of α Tubulin protein expression in the nuclear extract of Oli neu cells, differently from the cytoplasm extracts reported in Figure 4A The relative molecular weight is on the left.
