# Supplementary material for: Effects of Selective α7 Nicotinic Acetylcholine Receptor Stimulation in Oligodendrocytes: Putative Implication in Neuroinflammation
Source: Cells. 2025 Jun 20;14(13):948. doi: 10.3390/cells14130948 (PMC12248911; doi:10.3390/cells14130948)
Supplement: Supplementary file 1 [file cells-14-00948-s001.zip › cells-3449469-supplementary.pdf]

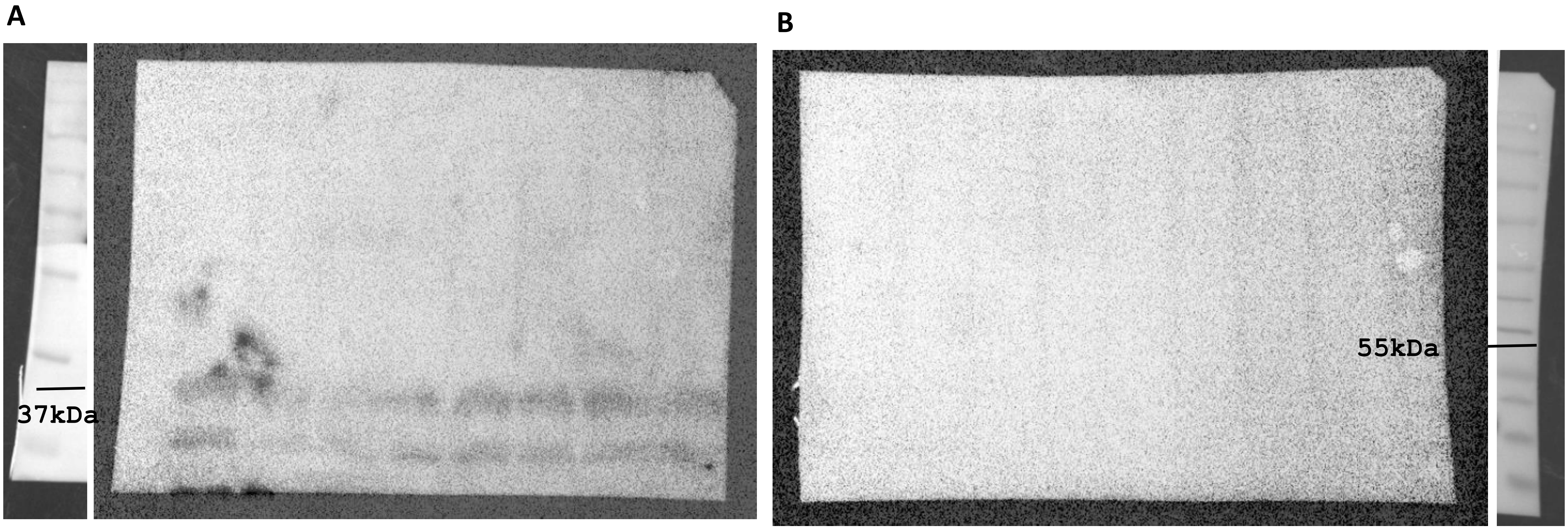

Supplementary Figure 1. (A) Representative Western blot showing the faint expression of Histone H3 protein in the cytoplasm extracts of Oli-neu cells, to compare with the nuclear extracts in Fig. 4B . The relative molecular weight is on the right. (B) Representative Western blot shows the absence of α-Tubulin protein expression in the nuclear extract of Oli-neu cells, differently from the cytoplasm extracts reported in Fig. 4A. The relative molecular weight is on the left.
